# Augmented Renal Clearance in Severe Post-traumatic Acute Pancreatitis: A Case Report From the Intensive Care Unit of Treichville University Hospital

**DOI:** 10.7759/cureus.93863

**Published:** 2025-10-05

**Authors:** Servais Sontia Sai, Kouassi Henri Ahue, Morell Cecinette Adjo, Koffi Isidore Kouamé, Ngolo Adama Coulibaly, Narcisse Boua

**Affiliations:** 1 Intensive Care Unit, Treichville University Hospital, Abidjan, CIV; 2 Faculty of Medicine, Université Félix Houphouët-Boigny, Abidjan, CIV; 3 Digestive and Endocrinology Surgery Unit, Treichville University Hospital, Abidjan, CIV; 4 Intensive Care Unit, Yopougon University Hospital, Abidjan, CIV

**Keywords:** abdominal trauma, critical care medicine, renal hyperclearance, severe acute pancreatitis, therapeutic failure

## Abstract

Acute kidney injury (AKI) represents one of the most prevalent organ dysfunctions encountered in severe acute pancreatitis. Augmented renal clearance (ARC) is an emerging phenomenon in intensive care medicine, with younger age and traumatic injury recognized as significant predisposing factors. In contrast to AKI, this pathophysiological entity has not been previously documented in the context of severe acute pancreatitis. Herein, we present a case of a 20-year-old female who sustained blunt abdominal trauma resulting in hemoperitoneum consequent to hepato-pancreatico-renal injury. The postoperative course following initial exploratory laparotomy was complicated by secondary bacterial peritonitis and subsequent septic shock. Despite appropriate antimicrobial therapy and supportive care, persistent sepsis prompted consideration of ARC, subsequently confirmed through pharmacokinetic assessment. Therapeutic optimization of antibiotic dosing protocols yielded clinical improvement and successful resolution. This case report illustrates the potential occurrence of ARC in the setting of severe acute pancreatitis and highlights the importance of pharmacokinetic monitoring in critically ill patients.

## Introduction

Acute kidney injury (AKI) is the most well-described renal dysfunction in the ICU. Recently, the concept of augmented renal clearance (ARC), initially observed in kidney transplant recipients, has also been described in intensive care. It is defined as an increase in renal creatinine clearance above 130 mL/min. It is observed in young patients, burn victims, and trauma patients [1,2]. In our region (Sub-Saharan Africa), ARC is little known among practitioners.

Post-traumatic pancreatitis is a rare condition [3,4]. Regardless of its etiology, these pancreatitis cases often progress to AKI [5]. ARC can also occur following trauma [6,2], but it has never been described in the course of post-traumatic severe acute pancreatitis (SAP).

We report a case of ARC in a 20-year-old female patient who developed acute pancreatitis in a traumatic context complicated by sepsis. The objective is to draw attention to the possible occurrence of this complication during traumatic acute pancreatitis.

## Case presentation

**Figure 1:**
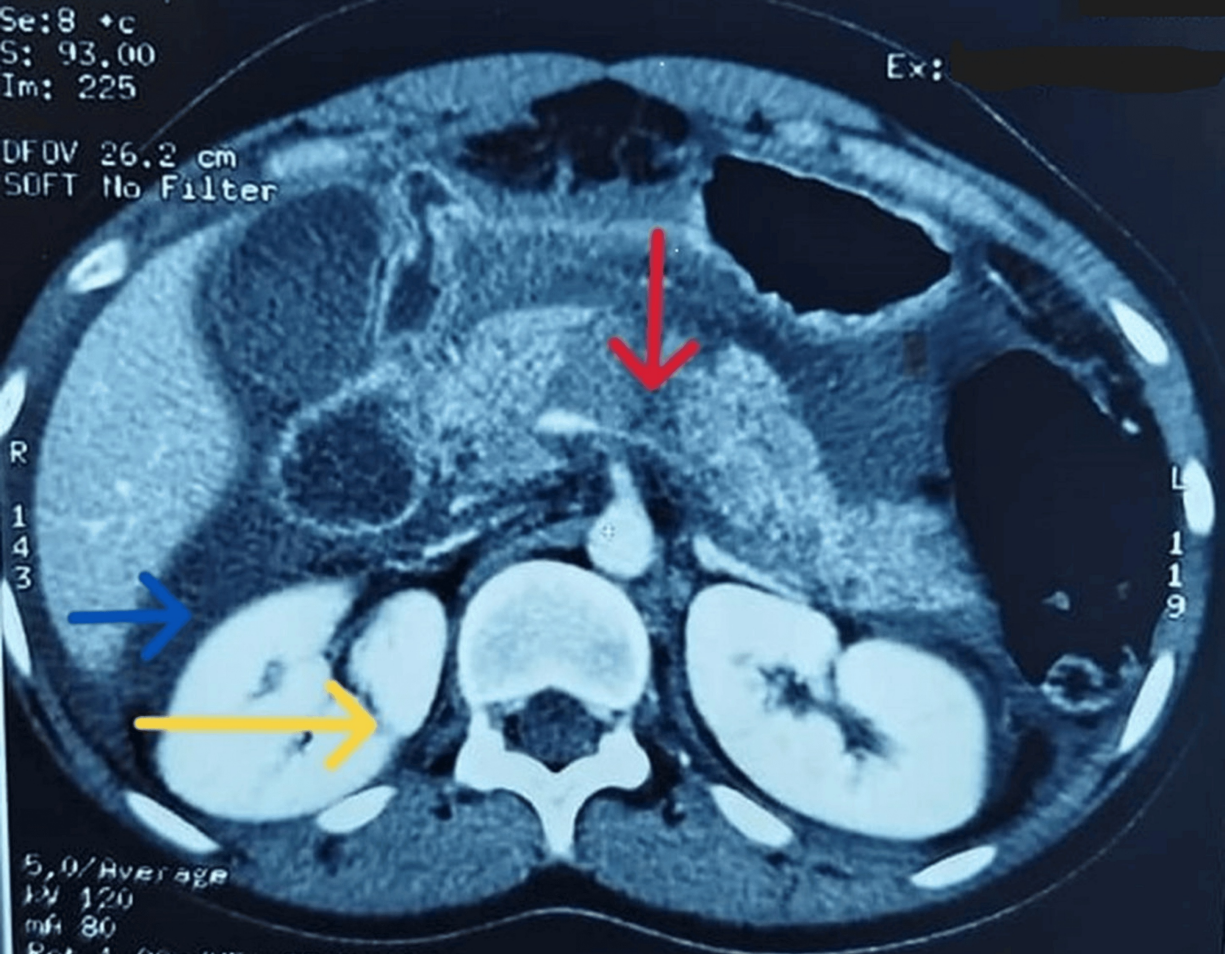
Abdominal CT scan of a hemoperitoneum related to the right hepato-pancreatico-renal contusion. Red arrow: corporo-isthmic pancreatic hypodensity consistent with contusion. Blue arrow: hemoperitoneum. Yellow arrow: discontinuity of the right kidney.

A 20-year-old female patient suffered from a neglected abdominal trauma during a recreational activity (karting session). Admitted to the emergency department two days after the accident, the initial examination revealed an abdominal contusion in an afebrile patient with stable cardiorespiratory status. Abdominal CT scan revealed a hemoperitoneum related to right hepato-pancreatico-renal contusion (Figure 1). An emergency laparotomy performed the same day confirmed the CT scan findings (with hemoperitoneum, edema of the isthmic portion of the pancreas with disseminated candle-wax appearance lesions, and a grade 2 Moore hematoma of liver segment 3). Hepatic suturing and peritoneal lavage were performed.

On the 12th postoperative day, the patient developed secondary peritonitis (associated with evisceration and hemodynamic instability), requiring surgical revision following hemodynamic resuscitation. A second surgical exploration revealed suppuration of the abdominal wall with concomitant involvement of the main pancreatic duct (Wirsung duct). A microbiological sample was obtained intraoperatively, and the surgery consisted of watertight repair of the pancreatic duct, necrosectomy, and complete peritoneal lavage. The patient was admitted to the intensive care unit following this second surgical procedure, and clinical assessment concluded septic shock (refractory hypotension despite fluid resuscitation, mild fever, confusion, and deterioration of general condition). Management in intensive care, therefore, consisted of adding vasopressor therapy (noradrenaline) and empirically adjusting the anti-infective treatment: discontinuation of ceftriaxone, administration of amikacin (two doses of 1.5 g given 24 hours apart), and piperacillin-tazobactam (12 g/24 hours by continuous infusion). We subsequently added fluconazole (400 mg/12 hours) based on the results of infectious specimens (*Escherichia coli* and *Candida albicans*, both susceptible to piperacillin-tazobactam and fluconazole, respectively). After 72 hours of appropriate treatment, we observed persistent septic shock (continued requirement for noradrenaline and persistence of infectious signs). Given the persistence of sepsis despite adapted treatment and considering the traumatic context, the hypothesis of ARC was raised. We therefore measured creatinine renal clearance (CrCl), estimated using the following formula on a 24-hour urine collection: (urinary creatinine) × (urinary volume) / (plasma creatinine). This approach confirmed the diagnosis with a CrCl = 192 mL/min. Detailed laboratory values are presented in Table 1.

**Table 1:** Laboratory parameters at the emergency department (ED) admission, day one, and day 15 of intensive care unit admission. * ED values correspond to values at the emergency department admission; day 1 in ICU corresponds to ICU admission; day 15 represents the follow-up assessment during the ICU stay. ** Creatinine clearance is the product of urinary creatinine and urine output divided by plasma creatinine. WBC: white blood cells; NA: not available.

Laboratory parameters	Units	*ED values	*Day 1 in ICU values	*Day 15 in ICU values	Reference range
Urinary creatinine	mg/L	NA	1.093	1.961	600-1.800
Urine output	mL/min	NA	1.23	1.44	1-2
Plasma creatinine	mg/L	9	7	8	6-11
**Creatinine clearance	mL/min	NA	192	352.98	<130
WBC count	cells/mm³	11,450	28,320	12,340	4,000-10,000
Hematocrit	%	29.9	28.7	31.1	36-46
Hemoglobin	g/dL	11.4	8.9	10.2	12-16
Blood urea nitrogen	g/L	0.26	0.31	0.28	0.12-0.40
Lactate	mmol/L	2.1	4.8	2.2	0.5-2.0
C-reactive protein	mg/L	96	156	48	<10

Unable to perform therapeutic drug monitoring of antibiotics, we optimized the dose of piperacillin-tazobactam to 24 g per day. The evolution was favorable from the 48th hour, with weaning from vasoactive amines, regression of infectious markers (CRP, leukocytosis), and improvement in the general condition. The patient was transferred to the surgical unit after a 15-day stay in the ICU. A new measurement of creatinine clearance performed upon discharge from the ICU noted an accentuation of augmented clearance (clearance = 352.98 mL/min). Multidisciplinary follow-up involving nephrologists, infectious disease specialists, and surgeons was conducted upon her discharge.

## Discussion

We report a case of ARC complicating SAP following abdominal contusion, a previously unreported outcome in post-traumatic pancreatitis. This observation highlights the potential occurrence of ARC in traumatic pancreatitis and discusses its under-recognition in our region, pathophysiological mechanisms, and clinical implications.

From an epidemiological perspective, the occurrence of pancreatic injury is rare (0.2-6%) during abdominal contusions [7,8]. These post-traumatic pancreatic lesions progress to acute pancreatitis in only 2% to 6% of cases [4]. As observed in our case, it is the presence of ductal injury that determines the risk of developing pancreatitis [7]. Beyond its rarity, this etiology appears to be underestimated in low-income countries, where access to abdominal computed tomography may be difficult. Regarding ARC, it is a recently described concept in intensive care and is still poorly documented in our region (Sub-Saharan Africa). It is a renal dysfunction that manifests as renal hyperfunction. It contrasts with AKI, which is the best-described renal complication in intensive care (due to its high frequency) and is also one of the most feared organ failures in SAP (10% to 70% of cases) [5].

Post-traumatic SAP and ARC are two conditions whose diagnosis is not straightforward in tropical settings. The possibility of normal pancreatic appearance on early initial CT scan, as well as the existence of a latent interval (between trauma and onset of pancreatitis: 4-15 days), may lead to a missed diagnosis of this lesion [7]. In our patient, the pancreatic injury was detected on admission to the hospital (day three post trauma) through abdominal CT scan. However, lipase measurement was only performed during the intensive care stay (day 15 after the first surgical intervention). As for ARC, its diagnosis relies on the biological measurement of renal creatinine clearance exceeding 130 mL/min/1.73 m². In the absence of specific clinical signs, this measurement is primarily initiated based on presumptive arguments (suggestive context). Thus, the best-identified predisposing factors to date are young age, trauma, burns, absence of AKI, and absence of significant comorbidities [9]. This diagnosis, therefore, requires particular attention and knowledge of these risk factors. The occurrence of ARC in our patient appears to have been favored by her young age, the traumatic context, and the sepsis.

The prevalence of ARC in the ICU is estimated between 29% and 39% and varies considerably according to the type of patients admitted to the ICU. In neurological intensive care units and trauma intensive care units, this prevalence can reach 74% and 58% respectively. However, it does not exclusively concern subjects in intensive care, as it is also observed in one-third of patients who have undergone abdominal surgery (30% in surgical hospitalization units) and also in those who have experienced trauma (35%) [10]. It may be observed over a given period or persist throughout the patient's hospital stay [11]. In our case, the time of onset of ARC remains unknown, but it certainly occurred before ICU admission. Moreover, it persisted until ICU discharge (27th post-traumatic day).

The primary consequence of ARC is pharmacokinetic, with enhanced renal elimination leading to reduced plasma concentrations and potential therapeutic failure. Standard antibiotic dosing in ARC patients risks treatment failure [12,13]. In our case, the postoperative peritonitis and persistent infection following the second laparotomy were likely attributable to subtherapeutic antibiotic concentrations secondary to ARC, prompting dose escalation of piperacillin-tazobactam to 24 g/day. However, the causality between dose adjustment and clinical improvement remains uncertain, as recovery may have resulted from ongoing surgical drainage, delayed antimicrobial efficacy, or spontaneous infection resolution.

## Conclusions

Given the concurrent presence of sepsis and trauma in our patient, we cannot establish a definitive causal relationship between ARC and acute pancreatitis. Until further cases are reported, clinicians should remain vigilant to the potential occurrence of ARC in inflammatory states (sepsis, trauma, burns, etc.). Early detection of this complication and therapeutic adjustment of renally eliminated drugs should be undertaken to prevent complications related to therapeutic failure.
